# Auditory and visual systems organization in Brodmann Area 8 for gaze-shift control: where we do not see, we can hear

**DOI:** 10.3389/fnbeh.2013.00198

**Published:** 2013-12-10

**Authors:** Marco Lanzilotto, Vincenzo Perciavalle, Cristina Lucchetti

**Affiliations:** ^1^Section of Physiology and Neuroscience, Department of Biomedical Sciences, Metabolic and Neuroscience, University of Modena and Reggio EmiliaModena, Italy; ^2^Section of Polyclinic, Interdepartmental Facilities Center, University of Modena and Reggio EmiliaModena, Italy; ^3^Section of Physiology, Department of Biomedical Sciences, University of CataniaCatania, Italy

**Keywords:** FEF, PEEF, auditory system, visual system, oculomotor system, gaze shift, monkey

Hearing is especially important for most primate species as they live in habitats of dense vegetation that limits vision. Stebbins (1980) summed up the evolution of the auditory system by assuming that earliest mammals exploited nocturnal niches since they were relatively free of many of the large, diurnal, predacious reptiles. Therefore, hearing and smell were more useful at night than vision.

Our vision is limited not only in the dark but also outside the visual field. In fact, if we observe the behavior of a predator like a feline, oriented toward its prey, and at the same time a sound occurs behind, we might note three principal different behaviors: the predator could maintain its gaze and ears on the prey neglecting the sound source; the predator could maintain its gaze on the prey rotating ears and then shifting its auditory attention toward the sound source; finally the predator could break its attention and orient gaze and ears toward the sound source. A similar behavior is seen in human beings during social interaction with two or more interlocutors.

In humans, orienting movements are carried out by the eyes, head, and/or body operating alone or in various combinations depending on the behavioral situation. However, in non-human primates, such as macaque monkeys, head orienting movements and, more generally, gaze-shift are accompanied by ear orienting movements, which allow the shifting of auditory attention toward a sound of interest (Bon and Lucchetti, 1994, 2006; Lucchetti et al., 2008; Lanzilotto et al., 2013; Yin, 2013).

Considering all these assumptions, the auditory system could have an important role to detect information even from regions of the space that the visual system cannot explore without orienting movements. In other words, where we cannot see, we can hear.

Through this opinion article, we argue that Brodmann Area 8 receives information from both auditory and visual systems and organizes a transformation of these sensory signals into gaze-shift motor commands. Our hypothesis is that this sensory-motor transformation is spatially organized, from both anatomical and functional points of view.

Anatomical and functional properties of the Brodmann Area 8 (consisting in Area 8A plus Area 8B) support a medio-lateral organization for both auditory and visual systems. In particular, the lateral portion, corresponding to Area 8A or Frontal Eye Field (FEF), could play a role in receiving visual and auditory information from a central part of the visual field and then in organizing gaze-shift motor commands toward it. Otherwise, the medial portion, corresponding to Area 8B or Premotor Ear-Eye Field (PEEF), could play a role in receiving principally auditory information from a peripheral region of the space and then in organizing gaze-shift motor commands toward it.

## Frontal eye field

The (FEF, area 8A) is a functional field in the prefrontal cortex, located in the rostral bank of the arcuate sulcus of macaques. FEF participates in the transformation of visual signals into saccade motor commands (Schall, 1997; Sato et al., 2001). FEF is innervated in a topographic fashion by areas of both the dorsal and ventral visual streams originating in extrastriate visual cortex (Schall et al., 1995). The ventral part of the FEF receives visual information from areas where fovea is clearly represented and retinotopically organized, such as MT and V4; from areas in the inferotemporal cortex such as TEO and caudal TE involved in central vision; finally from areas in parietal cortex having poor retinotopic organization but important for oculomotor transformation, such as LIP. In support of anatomical evidence, Suzuki and Azuma (1983) found that neurons located in the ventral FEF had relatively small visual receptive fields (RFs) representing the foveal and parafoveal regions. Moreover the ventral FEF works for generating short amplitude saccades (Bruce et al., 1985). On the contrary, the dorsal part of FEF is connected with retinotopically organized areas where peripheral visual field is represented, such as areas MSTd and PO, located medially, that are involved in peripheral vision, as well as from LIP. In support of anatomical evidence, Suzuki and Azuma (1983) found that neurons located in dorsal FEF had larger RFs and eccentric from the fovea. Finally, the dorsal FEF is responsible for generating larger amplitude saccades (Bruce et al., 1985).

Otherwise, it is well known, that FEF receive also information from the auditory system, participating thus, in the transformation of auditory signals into motor commands. An interesting study show in fact as FEF, in human, is a multimodal area involved in ultra-rapid responses for auditory and visual stimuli (Kirchner et al., 2009).

The auditory cortical system is constituted by two different streams, termed dorsal and ventral, projecting to the frontal cortex in non-human primates (Romanski et al., 1999a,b). The dorsal stream directly projects from caudal auditory belt to area 8A (Romanski et al., 1999a), bringing information about sound spatial localization. The ventral stream, involved in codifying the features of the auditory stimulus, originating from rostral auditory belt and rostral auditory parabelt, is connected indirectly to area 8A passing through the ventral prefrontal cortex (Romanski et al., 1999a; Gerbella et al., 2010; Rauschecker and Romanski, 2011).

In accord to anatomical evidence, many neurons in FEF respond to auditory stimuli. FEF has an interesting medio-lateral gradient regarding both the auditory spatial selectivity and proportion of auditory neurons. In particular, Azuma and Suzuki (1984) showed that in in the ventral part of FEF, auditory neurons have RFs located at 10° respect to the azimuth. As one moves more medially toward dorsal FEF, auditory neurons have more eccentric RFs till 40° respect to the azimuth. Moreover, in dorsal FEF the number of auditory neurons gradually increases while the number of visual neurons gradually decreases.

## Premotor ear eye field

Recently, area 8B, located medially to FEF, has been renamed as a new frontal field: PEEF (Lucchetti et al., 2008; Bon et al., 2009; Lanzilotto et al., 2013). PEEF is characterized by having principally connections with the auditory system, and has been hypothesized to participate in the transformation of auditory signals into motor commands. In particular, PEEF is connected directly to the caudal auditory belt through the dorsal auditory stream (Romanski et al., 1999a), which has a role for sound spatial localization. In accord to these connections, three principal classes of neurons have been identified in PEEF: auditory neurons, auditory-motor neurons, and motor neurons. The auditory stimuli that better elicit a response in PEEF's neurons are complex environmental stimuli as experimenter voices, while ear and/or eye movements represent the motor effectors controlled by this region. For these reasons, PEEF has been proposed as a new field having a possible role to detect auditory stimuli in the space through ear and eye movements (Lanzilotto et al., 2013).

## Conclusion

**Figure 1:**
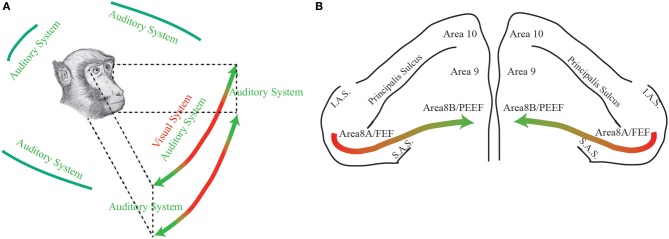
**(A)** The figure represents the space detected by primates through two different sensory modalities: green, auditory modality; red, visual modality. **(B)** Dorsal view of the monkeys' prefrontal pole. The colored arrow represents the functional gradient discussed in the paper. Area 8A or FEF: Frontal Eye Field; Area 8B or PEEF: Premotor Ear-Eye Field; Area 9; Area 10; IAS: Inferior Arcuate Sulcus; SAS: Superior Arcuate Sulcus.

Through this opinion article, in light of PEEF discovery (Lucchetti et al., 2008; Bon et al., 2009; Lanzilotto et al., 2013), we propose that the part of the frontal cortical region, consisting of area 8A (FEF) plus area 8B (PEEF), could be thought having a medio-lateral organization devoted for the exploration of different regions of the space. In particular, the lateral part—which receives visual information from retinotopically organized areas and has auditory neurons with RFs close to the azimuth—could have an important role in receiving visual and auditory information from a central region of the space and then in organizing eye motor commands toward it. On the other hand, the medial part—which receives, principally, auditory information through the dorsal auditory stream—could have an important role in receiving auditory information from a peripheral region of the space and then in organizing ear-eye motor commands toward it (Figure 1).
